# Supplementary material for: Estimating the incidence of dengue in international air travelers from non-endemic countries between 2010–2019
Source: PLoS Negl Trop Dis. 2025 Jul 9;19(7):e0013291. doi: 10.1371/journal.pntd.0013291 (PMC12279095; doi:10.1371/journal.pntd.0013291)
Supplement: S1 Text — Detailed methods and Supplementary Figures. (DOCX) [file pntd.0013291.s001.docx]

**Supplementary Methods**

*Mathematical model*

Analysis 1 (Estimating total infections and cases): To estimate the total number of dengue infections and cases among all 43 origin countries, we applied the following formula. Let $N_{jk}^{t}$ be the number of trips taken from country *j* to country *k* in year *t*, $D_{jk}^{t}$ be the average trip duration in days, and $\lambda_{k}$ be the annual dengue FOI in country *k* with log standard deviation $\sigma_{k}$, (i.e., individuals who are fully susceptible to dengue become infected at a rate $\lambda_{k}$). The expected number of dengue infections $i_{jk}^{t}$ in year *t* among travelers from country *j* to country *k* is:

$$E\left( i_{jk}^{t} \right)=N_{jk}^{t}\left[ 1-\exp\left( -\lambda_{k}\frac{D_{jk}^{t}}{365} \right) \right]\approx N_{jk}^{t} \lambda_{k}\frac{D_{jk}^{t}}{365} (1)$$

since trip duration is short, and $\lambda_{k}\frac{D_{jk}^{t}}{365}$ is small. We did these calculations for each combination of origin country (j), destination country (k), and year (t). The expected number of dengue cases $E\left( s_{jk}^{t} \right)=p_{s}E\left( i_{jk}^{t} \right)$, where $p_{s}$ is the proportion of infections that are symptomatic. The total annual number of travel-associated dengue cases by origin country is calculated as $E\left( s_{j}^{t} \right)=\sum_{k} E\left( s_{jk}^{t} \right)$. To account for uncertainty in $\lambda_{k}$ and $p_{s}$, we generated 10,000 bootstrap realizations from the distribution of $\Lambda_{k}\sim N\left( \lambda_{k},\sigma_{k} \right)$ and $\log\left( \frac{1-p_{s}}{p_{s}} \right)\sim N(\log\left( 4.3 \right),\log\left( 2.8 \right))$(68). Bhatt et al(68) reported the mean and standard deviation of the global inapparent to apparent ratio (i.e. the odds of having inapparent infection), so we assumed that the log-odds was normally distributed with variance chosen to recover a standard deviation of 2.8 on the odds scale.

Analysis 2 (Estimating case reporting fractions by origin country): We estimated the proportion of estimated cases that were reported by using negative binomial regression to analyze case data from countries that reported travel-associated cases regardless of destination country. If $\rho_{j}^{t}$ is the proportion of dengue cases that are reported to surveillance systems in origin country *j* in year *t*, then the expected number of reported cases (${cr}_{j}^{t}$) in origin country *j* in year *t* is given by:

$$E\left( {cr}_{j}^{t} \right)=\rho_{j}^{t}E\left( s_{j}^{t} \right)\approx\rho_{j}^{t}\sum_{k} N_{jk}^{t}\frac{D_{jk}^{t}}{365}\lambda_{k} (2)$$

As $\log\left( E\left( {cr}_{j}^{t} \right) \right)=\log\left( \sum_{k} N_{jk}^{t}\frac{D_{jk}^{t}}{365}\lambda_{k} \right)+\log\left( \rho_{j}^{t} \right)$, $\rho_{j}^{t}$ was estimated as a fixed effect in a negative binomial regression model without an intercept, and with $\log\left( E\left( s_{j}^{t} \right) \right)$ as an offset term. To propagate uncertainty from the endemic FOI and symptomatic probability, we performed this regression on each bootstrap sample from Analysis 1.

Analysis 3 (Estimating infection reporting fractions by origin and destination country): We examined patterns in reporting by origin and destination country using case data from countries that reported the locations where travelers were infected. To have a reported case with known country of infection, an individual must: A) seek care and get tested, which may depend on both the origin country (e.g., due to differences in seeking care or healthcare resources) and destination country (e.g., clinicians may be more likely to test a febrile returned traveler for dengue if the destination was one that is widely known as being dengue endemic), and B) have the country of infection determined (which may also depend on both the origin and destination country). These probabilities are not identifiable when information on the country of infection is missing except under strong assumptions that they are constant across all countries. Therefore, we restricted analyses to reported cases of known destination and to years in which countries reported country of infection. We then estimated the fraction of infections acquired in destination countries that are reported and have a known country of infection.

If $\alpha_{jk}^{t}$ is the fraction of total dengue infections acquired by travelers from origin country *j* in destination country *k* that are reported and have known country of infection, then the expected number of reported cases $c_{jk}^{t}$ in year *t* with known country of acquisition among travelers returning from country *k* to country *j* is:

$$E\left( c_{jk}^{t} \right)=\alpha_{jk}^{t}N_{jk}^{t}\left[ 1-\exp\left( -\lambda_{k}\frac{D_{jk}^{t}}{365} \right) \right]\approx\alpha_{jk}^{t} N_{jk}^{t} \lambda_{k}\frac{D_{jk}^{t}}{365} (3)$$

In words, equation 3 states that we estimated the number of reported travel-associated dengue cases with known country of acquisition by multiplying the result from equation 1 by the fraction of dengue infections among travelers that are reported and have known country of acquisition. We did these calculations for each combination of origin country (j), destination country (k), and year (t). To incorporate uncertainty in $\lambda_{k}$ into our estimates of $\alpha_{jk}^{t}$, we maximized the Poisson likelihood directly using Bayesian MCMC as detailed below. Finally, for some countries the number of cases reported for a country/year combination was reported as ≤5. In this case, we assumed that three cases were reported in that country/year combination since this is the average of a uniform distribution with minimum and maximum values of 1 and 5 respectively. In a sensitivity analysis, we assumed that one case was reported in that country/year combination. We maximized the likelihood using Bayesian MCMC, implemented using the package rstan(74). We ran 4 chains with 2,000 iterations each, discarding the first 1,000 as burn-in and thinning by a factor of 10, to produce posterior parameter distributions with 400 samples. To incorporate uncertainty in the GDTM estimates of $\lambda_{k}$, we used a uniform prior $U(\lambda_{k}e^{-\sigma_{\lambda_{k}}},\lambda_{k}e^{\sigma_{\lambda_{k}}})$, where $\sigma_{\lambda_{k}}$ is the estimated standard deviation of $\lambda_{k}$ from the GDTM. For the reporting fraction $\alpha_{jk}^{t}$ we used an uninformative prior $U(0,1)$.

*Model output and assessing model fit*

We fitted four models for how reporting fractions varied across time and by origin and destination country using data from 22 origin countries that reported information on country of infection for at least one year between 2010 and 2019, for a total of 12,272 country-pair-years. First, we varied whether the country-specific reporting probability was assumed to be constant ($\alpha_{jk}^{t}=\alpha_{jk}$) over the period 2010-2019 or time-varying ($\alpha_{jk}^{t}\neq\alpha_{jk}$) (“time-constant” reporting vs. “time-varying” reporting). Second, we compared results in which the reporting probability for a pair of countries was driven by characteristics of the origin and destination countries without any interaction ($\alpha_{jk}^{t}=\alpha_{j}^{t}\alpha_{k}^{t}$) to results where we fitted separate reporting probabilities for each pair of countries ($\alpha_{jk}^{t}\neq\alpha_{j}^{t}\alpha_{k}^{t}$) (“no interaction” model vs. “pairwise” model). Finally, we assessed a model in which we made both assumptions, leading to four models in total for country-pair reporting probabilities: i) time-constant and no interaction, ii) time-varying and no interaction; iii) time-constant and pairwise; iv) time-varying and pairwise. A model with time-varying reporting probabilities varying pairwise by origin and destination country (“time-varying, pairwise”) was well supported by the data, relative to less parsimonious models (difference in expected log pointwise predictive density 505 (standard error 333) compared to the next best fitting model). We present results from this model. A trace plot of the log-posterior is shown in Figure S5, demonstrating adequate mixing of chains.

Finally, using the time-varying and no interaction model above as the pairwise model cannot be used to make predictions about held-out pairs, we performed a leave-one-out validation to understand patterns in which country pairs showed poor goodness of fit or were otherwise inconsistent with other country pairs. We removed one country-pair (all years) from the data and fit the model to the remaining data. We predicted cases by year for that country pair and compared these predictions to the observed reported cases. We performed this validation for the 100 country pairs with the highest average annual reported cases. We fitted linear regression lines to the predicted vs. observed case numbers and defined outliers as points with Cook’s distance greater than four times the average; Cook’s distance measures the influence of removing a single point. In this analysis, points that lay above the regression line indicated that more cases were predicted than observed, meaning that travelers in that country pair were at lower risk than the endemic FOI predicted or that reporting probabilities in travelers from that country pair were lower than average.

Figure Legends

Figure S1: Location-specific FOI estimates from Vicco et al.(55) (black) and national, population-weighted FOI estimates and 95% CI from the GDTM (red)

Figure S2: Reported total cases 2010-2019 by origin country against predicted annual cases

Figure S3: Estimated fraction of dengue cases reported to national surveillance systems between 2010-19 among 34 origin countries reporting dengue cases, accounting for uncertainty in endemic FOI and probability of symptoms.

Figure S4. Annual observed and predicted reported cases from leave-one-out validation, assuming constant FOI in endemic countries. The country pairs with the 100 highest total reported cases from 2010-2019 were left out one at a time, and reported cases by year were predicted from a model fit to the remaining data. Outliers are labeled in red

Figure S5: Markov chain Monte Carlo trace plot of log-posterior from time-varying and pairwise model
