# Supplementary material for: Estimating the incidence of dengue in international air travelers from non-endemic countries between 2010–2019
Source: PLoS Negl Trop Dis. 2025 Jul 9;19(7):e0013291. doi: 10.1371/journal.pntd.0013291 (PMC12279095; doi:10.1371/journal.pntd.0013291)
Supplement: Attachment — Submitted filename: Review.docx [file pntd.0013291.s013.docx]

This study is relatively short and straightforward and estimates travel-associated dengue cases by country. It is well written and the analysis, at least from my perspective, appears adequate. I therefore don’t have any major comments and relatively few minor comments.

Minor comments

Line 60: ‘arbovirus pathogen’… ‘to cause disease in humans’ (?)

Line 67: Just a thought really, the emphasis here is on the potential use of this information to inform messaging for travellers and clinicians, but is there also a society-level message here? There is a clear call from climate scientists and some global health researchers regarding the need to reduce air travel and here, there could be an additional argument for that with respect to reducing the speed of pathogen spread?! <https://doi.org/10.1016/j.gloenvcha.2020.102194>  [10.1016/S0140-6736(19)32680-7](https://doi.org/10.1016/S0140-6736(19)32680-7) <https://doi.org/10.1016/j.ecolecon.2021.107297> <https://stay-grounded.org/>

Line 78: Rephrase this sentence as it states ‘…are used to infer infection risk, which can provide estimates of infection risk’.

Line 112-113: ‘as a sensitivity analysis…’ may just be the way it’s phrased but it’s not clear how keeping something constant is a sensitivity analysis? Not sure what this sentence is describing. Perhaps elaborate on this? Also related to line 226/ 277 of the results – not sure here what the motivation for reducing the endemic FOI was?

Fig 2: Estimated fraction of dengue cases reported to national surveillance – so in some cases there were more cases reported than estimated to have occurred - how do you have proportions > than 1 here? Perhaps elaborate on this in the results?

Line 286: Rather than re-stating the results here, in the first paragraph it would be better to give a clear take home message arising from these results.

Line 395: Can you elaborate on this – perhaps by giving an example of what information is currently given (e.g. in the US) and how your result would be beneficial in addition to existing messaging? Similarly, can you elaborate on how the results can guide public health preparedness? Or here do you mean raising awareness of the risks rather than specific guidance?

Line 401: With respect to under-reporting of cases – are these symptomatic or asymptomatic?
